# Health risk assessment on musculoskeletal disorders among potato-chip processing workers

**DOI:** 10.1371/journal.pone.0224980

**Published:** 2019-12-03

**Authors:** Sunisa Chaiklieng

**Affiliations:** 1 Department of Environmental Health, Occupational Health and Safety, Faculty of Public Health, Khon Kaen University, Khon Kaen, Thailand; 2 Research Center in Back, Neck, Other Joint Pain and Human Performance (BNOJPH), Khon Kaen University, Khon Kaen, Thailand; University of Valencia, SPAIN

## Abstract

Musculoskeletal disorders (MSDs) are the most common complaint among industrial workers. The potato-chip processing industry involves workers in repetitive activities leading to MSDs. This cross-sectional descriptive study aimed to assess MSDs health risk among potato-chip processing workers. It was conducted among 107 randomly sampled workers from a distribution like other groups exposed to similar ergonomics factors. A MSDs health-risk assessment produced a matrix of combined results based on a self-report questionnaire (5 levels) and an ergonomics risk assessment using RULA (4 levels). The self-reported MSDs questionnaire showed that workers had moderate to very high discomfort levels, i.e., 11.21% trunk, 9.35% lower limbs, 8.41% upper limbs and 4.66% for the neck. Ergonomic risks were found to be at a very high level, 77.57%, and high risk level, 19.63%. The combined matrix assessments showed that most workers were at moderate to very high MSDs risk, i.e., 43.92% trunk, 36.45% upper limbs, 32.71% lower limbs and 20.56% for the neck. This health risk matrix found a higher proportion of workers presenting with MSDs health risk compared with the musculoskeletal disorders self-assessment alone. Therefore, the MSDs risk matrix assessment could be useful for surveillance screening prior to implementing a risk-reduction program. Further, using ergonomics training programs and improving work stations for high-risk groups are also recommended based on the ergonomic and health risk assessments in this study.

## Introduction

Musculoskeletal disorders (MSDs) are the most common work-related illnesses globally. Each year, Thai workers suffer from occupational diseases that need treatment. In 2017 the Thai Worker Compensation Fund, Social Security Office reported MSDs as the leading cause of work-related illness of 27,395 Thai workers compensation cases. Lifting or handling heavy loads was the cause for 625 workers while working posture and lifting affected 2,757 workers [1]. From 2012–2016, the most common disorders involving MSDs were joint pain and inflammation of the muscles was reported at 17.65% [2]. Ergonomic factors involved in heavy physical work causing MSDs and physical fatigue were lifting and forceful movements [3], bending and twisting, and static work postures [4, 5].

Assessment of the likelihood of exposure to ergonomic risk factors can be made using ergonomics risk assessment and observation tools, such as the BRIEF’s survey to assess operator body movement and the ergonomic environment. In our previous study, the BRIEF’s survey was used to assess Hand-Operated Rebar Bender workers [6]. By observing posture, and movement of several parts of the body coupled with some exertion, such as lifting or moving material, REBA (Rapid Entire Body Assessment) was carried out among manual handling workers [7]. RULA (Rapid Upper Limb Assessment) by observing upper limbs and mainly static lower limb posture, has been used to measure ergonomic risk in repetitive use of upper limbs during work [8, 9]. Previous standards of risk assessment methods for repetitive hand exertions at high frequency were also OCRA (a concise index for the assessment of exposure to repetitive movements of the upper limbs), the American Conference of Governmental Industrial Hygienists (ACGIH) Threshold Limit Value for Hand Activity Level (TLV for HAL), and the Strain Index (SI) [10]. The strength of their association with musculoskeletal disorders (MSDs) was discussed before [11]. The TLV for HAL and SI use different constituent variables to quantify task physical exposures. Similarly, time-weighted-average (TWA), Peak, and Typical exposure techniques to quantify physical exposure from multi-task jobs make different assumptions about each task's contribution to the whole job exposure. Moore and Garg [12] recommended value of 5 as a threshold SI score to distinguish a safe job from a hazardous job, as means to assess jobs for risk of work-related musculoskeletal disorders of the distal upper extremities (hand, wrist, elbow).

Identification of severity of MSDs body fatigue commonly uses self-report interview questionnaires. The tool evaluates only the position of the body parts of interest as well as those in previous tools like the Standard Nordic Questionnaire (SNQ) predicting workers’ MSDs risk [13] can involve other self-report tools such as, the Cornell Musculoskeletal Discomfort Questionnaire (CMDQ) [14] or a questionnaire that describes the severity and the frequency of pain as previously used among garment workers [4].

MSDs can combine chronic conditions and acute injury situations as in the potato-chip processing industry. Those workers commonly use their upper limbs controlling all automated machines. It involves repetitive posture work with exertion at a periodic and regular interval. A basic health risk assessment (HRA) considers both the probability of exposure and the severity of an adverse health effect from that health hazards exposure [15]. However, the use of a single method of ergonomics risk assessment may not apply equally to all regional MSDs problems.

Not much is known about applying the two approaches for MSD prediction with a combination of two assessment methods developed for the health risk matrix. Combinations of worker’s self-report and the objective ergonomics assessment by the researcher's observations were developed as a new tool, in this study, for health risk assessment. The model was based on our previous studies conducted among industrial workers for predicting the risk of shoulder pain [15, 16]. This study of its kind using specified health risk assessment tools to assess the risk of MSDs among potato-chip processing workers. It has a potential for improving risk surveillance programs and an improvement plan for workstation ergonomics.

## Materials and methods

### Subjects

This cross-sectional descriptive study among potato-chip processing workers at a manufacturing site in Ayutthaya Province, Thailand was conducted between January and March 2017. The total workforce was 319 workers from 4 departments i.e. Cool room, Process, Packing, and Warehouse. Subjects were selected using simple random sampling based on a sample size calculation formula provided by Krejcie and Morgan [17]. The proportion estimated from a previous study on the health risk of back pain among industrial workers from medium to very high risk is 0.57 [18]. The desired level of confidence at 95% (*α* = 0.05) was 1.96 and under the precision of estimation in this study, e = 0.08), the minimal requirement of 103 persons in sample was calculated. The simple random sampling recruited subjects from each department by taking into consideration the total population of workers in each department and a distribution like other groups exposed to similar ergonomics factors. The final number of eligible and volunteer participants was 107 persons who met inclusion criteria; 1) Thai nationality, 2) at least six months work experience 3) worked with automated machinery for more than eight hours daily and sometimes handled materials. Exclusion criteria were; 1) had accident involved musculoskeletal surgery or treatment of chronic MSDs during the past year, 2) pregnant.

### Materials

#### 1) Questionnaires

The structured questionnaire previously used among industrial factory workers [15] included 4 parts, consisting of personal characteristics, health status, and work environment. The fourth part was a self-reported MSDs with 4 rating levels of the frequency (1–2 times per week, 3–4 times per week, once daily or every day, several time every day) and 4 rating levels of the severity of pains i.e., no pain, mild (annoying, interfering little to working), moderate (short pain interferes significantly to posture adaptation), severe (persistant pain disabling work ability), and very severe (persistant pain and unable to perform work and affecting quality of life). There were 4 anatomical regions of the body (neck, upper limbs (shoulders, forearms, hands/wrists), trunk (upper and lower back), lower limbs (legs, feet) for self–reporting in the past month [16]. The researchers summed multiplier scores from ranked frequency (4 levels) and ranked severity (4 levels) to produce discomfort scores (0–16) classified into 5 levels as shown below;

level 0 (score 0) = no discomfort
level 1 (score 1–2) = mild
level 2 (score 3–4) = moderate
level 3 (score 5–8) = severe
level 4 (score 9–16) = very severe

#### 2) Rapid Upper Limbs Assessment (RULA)

The Rapid Upper Limb Assessment (RULA) tool was used to assess ergonomics risk using the standard McAtamney & Corlett [8] observations of working posture. RULA comprised working posture of the upper limbs (forearms, wrists), neck, trunk, and legs. The researchers derived the final scores and ranked the ergonomics risk level as shown below in 4 levels;

level 1 (score 1–2) = low
level 2 (score 3–4) = moderate
level 3 (score 5–8) = high
level 4 (score 9–16) = very high

These RULA levels of ergonomics risk assessment were defined as, level 1, considered acceptable., level 2 should be checked and may need correction, level 3 should be checked and corrected as soon as possible and level 4 should be corrected immediately.

#### 3) Matrix of health risk assessment

A health risk assessment matrix (Table 1) was created to categorize the MSDs health risk assessment by the scores resulting from multiplying row scores of discomfort level by column scores of RULA assessed ergonomics risk level in the risk matrix [15]. The researchers derived the final scores and ranked the health risk assessment level among the workers as indicated below.

level 0 (score 0) = acceptable risk
level 1 (score 1–2) = low risk
level 2 (score 3–4) = moderate risk
level 3 (score 6–8) = high risk
level 4 (score 9–16) = very high risk

**Table 1:** Matrix of combined self-report discomfort level and RULA level derived MSDs risk scores.

MSDs risk		Level of ergonomics risk (RULA)			
		1	2	3	4
	**4**	4	8	12	16
	**3**	3	6	9	12
**Level of** **discomfort**	**2**	2	4	6	8
	**1**	1	2	3	4
	**0**	0	0	1*	2*

Remark: Colors simply notice the zone as the risk level from the calculated scores of that; green is an acceptable risk, yellow is low risk, orange is a medium risk, brown is high risk, and red is very high risk

*notice low risk of MSDs when workers rated no discomfort, and RULA indicated high to very high risk [15].

Results of MSDs health risk assessment were, level 0, an acceptable risk, level 1, also acceptable and low risk involving perceived slight pain and surveillance suggested. Level 2, moderate risk and control should be exhibited and training provided, level 3 high risk and measures should be taken to control and implement training or workstation modification and level 4 revealed a very high risk and immediate need for implementing safety management and engineering design [15].

### Data analysis

All data analyses were performed using STATA Version 10.0. Descriptive statistics were used to summarize personal characteristics, the levels of upper limb discomfort, the ergonomics risks, and the ergonomics health risks. Categorical variables were presented using frequency distribution and percentages and continuous variables were presented using mean (SD), median (min-max).

This study was reviewed and approved by the Khon Kaen university ethics committee on human research, Thailand (HE592309). All participants gave written informed consent before being enrolling in the study.

## Results

### Personal characteristics of potato- chip processing workers

Most workers were female (75.68%). The age range was 21–43 years and the average working period was 20.59 months. Most personnel had worked in the Packing Department, 66.36%, Process, 10.28%, Warehouse, 7.48%, and in the Cool room, 15.89%. Those percentages of workers also represented the proportion of workers among different units in this factory (30%-35% of workers in each Department).

Work characteristics in the Packing unit were carrying potato-chip ingredient (powder package) to fill the hopper, changing film roll for packaging, packing potato-chip bags in larger bags or cardboard boxes, package sealing. In the Cool room, worker’s tasks were inspecting/ grading of raw potato, driving a forklift, driving hand lift/control. In the Process Department, the main task was an inspection by cutting/ peeling potato with a knife. In Warehouse, workers’ tasks included pulling/holding cardboard boxes on conveyor/trolley, and handling packages onto pallets, or transporting pallets.

### MSDs risk by RULA

MSDs risk assessed by RULA revealed a very high level for 83 workers (77.57%), high risk for 21 workers (19.63%) and moderate risk for 3 workers (2.80%) as shown in Table 2.

**Table 2:** The number and percentage of potato-chip processing workers classified by level of ergonomics risk by RULA (n = 107).

Levels of ergonomics risk by RULA	Number (%)
**Low risk**	0 (0.00)
**Moderate**	3 (2.80)
**High**	21 (19.63)
**Very high**	83 (77.57)

All workers from the Department of Process (11 workers), Packing (71 workers) and Warehouse (8 workers) were at RULA high to very high risk (level 3 to level 4). For very high risk level required implementation, there were 7 workers (63.64%) who were from the Process Department, 61 workers (85.92%) from the Packing unit and 5 workers (62.50%) from the Warehouse Department. In Cool Room, 10 workers (58.82%) had very high risk, 4 workers (23.53%) were at high risk, and 3 workers (17.65%) had medium risk level.

### MSDs risk by self-report

MSDs risk assessed by self-report questionnaire showed moderate to very high risk level for 12 workers (11.21%) for the trunk, 10 workers (9.35%) for lower limbs, 9 workers (8.41%) for upper limbs, and 5 workers (4.66%) for the neck, as shown in Table 3.

**Table 3:** The number and percentage of potato-chip processing workers classified by level of self-report discomfort of MSDs (n = 107).

MSDs	Level of the discomfort of MSDs [Number (%)]				
	No discomfort	Mild	Moderate	Severe	Very severe
**Neck**	85(79.44)	17(15.89)	3(2.80)	1(0.93)	1(0.93)
**Upper limbs**	67(62.62)	31(28.97)	6(5.61)	2(1.87)	1(0.93)
**Trunk**	59(55.14)	36(33.64)	9(8.41)	2(1.87)	1(0.93)
**Lower limbs**	72(67.29)	25(23.36)	4(3.74)	4(3.74)	2(1.87)

### MSDs risk by a combined matrix of self-report and RULA

The combined MSDs risk assessment matrix showed that 47 workers were at moderate to very high risk level (43.92%) for the trunk, 39 workers (36.45%) for upper limbs, 35 workers (32.71%) for lower limbs and 22 workers (20.56%) for the neck. as shown in Table 4.

**Table 4:** The number and percentage of potato-chips processing workers classified by level of MSDs risk by combined matrix scores (n = 107).

MSDs	Level of HRA [Number (%)]				
	Acceptable	Low	Moderate	High	Very high
**Neck**	85(79.44)	0(0.00)	17(15.89)	3(2.80)	2(1.87)
**Upper limbs**	67(62.62)	1(0.93)	30(28.04)	6(5.61)	3(2.80)
**Trunk**	59(55.14)	1(0.98)	35(32.71)	9(8.41)	3(2.80)
**Lower limbs**	72(67.29)	0(0.00)	25(23.36)	4(3.74)	6(5.61)

## Discussion

### MSDs risk determined by RULA

The RULA ergonomics risk assessment found that all workers had higher than acceptable MSDs risk. In all workers, 77.57% were assessed at the very high level, followed by 19.63% at the high level, with 2.80% at the moderate level. This is consistent with electronics workers involved in repetitive behaviors, where workers had high and very high levels of ergonomics risk [18]. This may have occurred because potato- chip processing work requires constant physical movement and working with automatic machinery. Twisting also occurred due to the cramped work environment involving the inspection process with grading/ cutting raw potato and packing. Thus, most ergonomics risk levels were high, particularly in the packaging tasks. Similarly, the study of the repetitive and repeated upper limb movement involving automatic machinery in the production and assembly of electronic components by workers showed high level ergonomic risk, level 4 (high risk), especially in jobs requiring machinery [15]. Most workers were static standing with repetitive moving of upper limbs to hold task in front of automated machine or conveyer, our previous study indicated ergonomics risk factors by using tools of Rapid Entire Body Assessment (REBA) and RULA among the standing workers [16]. Most workers were at high to very high risk and there was a significantly linear correlation between risk level from REBA and RULA (r = 0.726, p-value<0.05) in the previous risk assessment [19]. In addition, the typical sewing worker’s ergonomics risk assessment using RULA was at risk level 3, in the textile industry, workers using sewing machines showed an increased ergonomics risk at levels 3 (high) to 4 (very high) because of working automatic machinery [20].

### MSDs risk determined by self-report

Self-reported musculoskeletal discomfort among potato- chip processing workers in the past month found that 44.86% of the staff reported their highest discomfort in the trunk (back), followed by the upper limb (36.45%) and the lower limbs (32.71%) and the neck. This was similar to a study of workers working with automatic machinery in the garment industry, where workers reported of pains at shoulders (44.9%), neck (40.8%) and back (34.7%), respectively [21]. Among sewing machine operators, the highest prevalence rates for musculoskeletal symptoms also involved the trunk, neck, and shoulders, respectively. The body parts most often affected by MSDs symptoms during the previous twelve months were upper limbs for both male and female workers. In addition, during the previous seven days- musculoskeletal symptoms reported also involved the upper limb and back for male workers in the footwear industry [22].

### Health risk

Information from objective RULA assessment showed that most workers were at a very high risk level (77.57%), and the others were at the moderate to high risk. However, subjective self-report assessment data showed that workers rated themselves at lower risk levels, i.e., only 11.21% were at moderate to very severe levels for the trunk, 9.35% for the lower limbs, 8.41% for the upper limbs and 4.66% for the neck. This apparent difference may be due to workers' habits of exertion or work activities. Workers are provided with at least one working break during the 4-hour work period, excluding lunch break. Moreover, some jobs involved rotating work to avoid repetitive work all day long. Workers’ safety behaviors, posture adjustments, breaks, and workstation adjustment could have affected their perception of musculoskeletal discomfort. However, when considering the risk of ergonomic work posture, the risks were mostly moderate to very high.

Combinations of both subjective and objective assessments in the risk matrix showed that 43.9% of workers were at moderate to very high-risk levels for the trunk, 36.45% for upper limbs, 32.71% for lower limbs and 20.56% for the neck. This evaluation showed that both results of ergonomics risk assessments (medium to very high risk of up to 100%) and self-report discomfort (mostly no discomfort to mild discomfort) perception were useful. The moderate health risk was at least the high level of ergonomics risk and mild discomfort. This was in line with the study that evaluated health risk assessment of shoulder, back and neck pain among call center workers [23].

Therefore, this risk matrix could be useful as a predictor model for surveillance programs for risk implication. Prevention of MSDs risk using ergonomics training programs and improving work stations among high-risk groups are suggested based on the ergonomics and health risk assessment in this study.

## Supporting information

S1 Dataset (XLSX)
